# Correlation-Based Anomaly Detection Method for Multi-sensor System

**DOI:** 10.1155/2022/4756480

**Published:** 2022-05-31

**Authors:** Han Li, Xinyu Wang, Zhongguo Yang, Sikandar Ali, Ning Tong, Samad Baseer

**Affiliations:** ^1^School of Information Science and Technology, North China University of Technology, Beijing 100144, China; ^2^Beijing Key Laboratory on Integration and Analysis of Large-Scale Stream Data, North China University of Technology, Beijing 100144, China; ^3^Department of Information Technology, The University of Haripur, Haripur 22620, Pakistan; ^4^School of Software, Dalian Jiaotong University, Dalian 116028, China; ^5^Department of Computer Systems Engineering, University of Engineering and Technology Peshawar, Peshawar 25000, Pakistan

## Abstract

In industry, sensor-based monitoring of equipment or environment has become a necessity. Instead of using a single sensor, multi-sensor system is used to fully detect abnormalities in complex scenarios. Recently, physical models, signal processing technology, and various machine learning models have improved the performance. However, these methods either do not consider the potential correlation between features or do not take advantage of the sequential changes of correlation while constructing an anomaly detection model. This paper firstly analyzes the correlation characteristic of a multi-sensor system, which shows a lot of clues to the anomaly/fault propagation. Then, a multi-sensor anomaly detection method, which finds and uses the correlation between features contained in the multidimensional time-series data, is proposed. The method converts the multidimensional time-series data into temporal correlation graphs according to time window. By transforming time-series data into graph structure, the task of anomaly detection is considered as a graph classification problem. Moreover, based on the stability and dynamics of the correlation between features, a structure-sensitive graph neural network is used to establish the anomaly detection model, which is used to discover anomalies from multi-sensor system. Experiments on three real-world industrial multi-sensor systems with anomalies indicate that the method obtained better performance than baseline methods, with the mean value of F1 score reaching more than 0.90 and the mean value of AUC score reaching more than 0.95. That is, the method can effectively detect anomalies of multidimensional time series.

## 1. Introduction

Internet of Things (IoT) is an extended network based on the Internet, which supports more intelligent physical object management. In recent years, a rapid growth of the IoT has been witnessed, and the number of connected IoT devices is estimated to exceed 500 million by 2030 [1, 2]; therefore, IoT-based services will appear on a large scale in different application domains [2]. In the era of “Industry 4.0,” digital twin, which is a “fusion of the real and the virtual entities,” has begun to use data such as sensors, physical models, and operational histories to represent the entire life cycle processes of physical entities [3]. In the actual production process, the digital twin entity is commonly established by monitoring one or more important, interdependent physical entities through sensors [4]. In general, the function of the sensor is relatively single, so a single sensor cannot fully reflect the physical world [5]. A multi-sensor system is a system that contains multiple sensors. A digital entity constructed by a multi-sensor system can reflect the entity and its behavior more comprehensively and can provide richer data for different kinds of analysis [6].

Hawkins [7] defined anomalies as data that are distinctive in a dataset, which is suspected not to be derived from random deviations but generated by a totally different mechanism. Anomaly detection refers to identifying anomalous data using various data processing models and techniques and is a prerequisite part for fault diagnosis [4]. Anomalies in the equipment or the environment, which reflect production problems, usually cause failures and economic losses and can even lead to catastrophic consequences [8]. Taking the electric power system as an example, we find that abnormal real-time monitoring of electric power and electrical equipment not only can obtain the running state of the equipment, but also will diagnose the fault in time. Therefore, finding anomalies can help to better ensure the safe and stable operation of a power and electrical equipment. On the contrary, failure to discover the anomalies implied in the sensing data will be unfavorable to the timely detection of problems of physical entities and may cause unnecessary losses. Thus, multi-sensor systems which can achieve data monitoring of complex equipment or an environment throughout its whole life cycle have become the main approach in the field of anomaly detection.

The collected data of a multi-sensor system is represented by multivariate time-series data, and different dimensions of the multivariate time-series data originate from different features of sensors. In complex industrial systems, anomalies are not always isolated. Due to the fuzzy physical interaction, small anomalies might spread between different sensors and gradually deteriorate into serious anomalies in some devices [9]. Taking the thermal power plant as an example, there are two devices: coal feeder and coal pulverizer. A coal feeder is responsible for conveying the coal to the coal pulverizer for crushing. In normal conditions, when the amount of coal conveyed by the coal feeder increases, the working load of the coal pulverizer also needs to increase accordingly in time, which indicates that the coal quantity of the coal feeder and the current of the coal pulverizer maintain a stable correlation. On the contrary, if the coal pulverizer is not adjusted within the allowable time range, it will cause coal blockage and insufficient output and even cause equipment damage and production interruption. That is, the complex correlation between sensors is closely related to anomalies.

In recent years, deep learning-based techniques have been used for the anomaly detection of multi-sensor systems. However, the potential relationships between sensors, which are helpful for finding anomalies, are not explicitly learned. That is, when the stable correlation is destroyed, it may indicate the occurrence of anomalies. Therefore, it is necessary to obtain the correlations between sensors in a multi-sensor system, and the main challenge of using these correlations in anomaly detection is that the functions of sensors vary greatly and the relationship between sensors changes dynamically, which requires dynamic discovery, representation, and detection of correlations. Hence, this paper proposes a correlation-based anomaly detection method of a sequential multi-sensor system, which learns a set of temporal correlation graphs from sensors and detects the deviations of these correlations. The proposed method involves two main stages. (1) Correlation-based graph model is constructed: Firstly, the time-series data generated by the multi-sensor system is divided according to the time window. Nodes and edges are, respectively, used to represent the features and the correlations between pairs of features. Then, temporal correlation graphs are constructed to represent the fluctuation of correlations between features in a sequential multi-sensor system. (2) Graph-based anomaly detection learns the anomaly detection model with a structured-sensitive graph neural network and then identifies the deviations from the learned correlations in the temporal correlation graphs.

To summarize, the main contributions of this work are as follows:

- This paper is a novel attempt to propose and construct the concept of temporal correlation graph by obtaining the correlation between the features in a multi-sensor system.
- A structured-sensitive graph neural network is used to learn the information in the temporal correlation graph, including attributes such as points, edges, and structure of the graph, and classify the graph based on the collected information for anomaly detection.
- The accuracy and stability of the proposed method are compared with baselines by conducting numerous experiments on several datasets, and hence accuracy and stability have proven better than those of the baselines.

The rest of this paper is organized as follows.  Section 2 briefly introduces the related work of anomaly detection.  Section 3 analyzes the correlation characteristics of the sensor data acquired by a multi-sensor system, defines the research problem, and describes the correlation-based anomaly detection method for multi-sensor system. The performance evaluation is given in Section 4, and Section 5 is the conclusion.

## 2. Related Work

An overview of the current state of anomaly detection is first presented. Then, the data-driven anomaly detection methods are reviewed. Since the proposed method is a deep learning method, the related work of machine learning methods and deep learning methods is also summarized.

### 2.1. Anomaly Detection

The most important aspect of quality monitoring in the industry is anomaly detection. With the application of multi-sensor systems in the industry, anomaly detection is starting to target more than just outliers. The data acquired by multi-sensor systems is reflected as high-dimensional time-series data with characteristics such as continuity and correlation. Therefore, the anomalies of physical entities characterized by multi-sensor systems are no longer reflected as a single outlier or small number of outliers, but through multiple anomalies of data with certain continuity. Currently, methods for anomaly detection can be broadly classified into two types, i.e., non-data-driven methods and data-driven methods [10].

### 2.2. Non-data-Driven Methods

The non-data-driven anomaly detection methods include physical model-based methods and signal processing-based methods. The former focus on obtaining the data signals on the system to be tested and analyzing the data processing results with the initially established model in order to obtain the abnormal diagnostic situation. The latter aims at investigating the techniques and methods of highlighting abnormal feature information.

However, the above methods require a priori knowledge and relevant equipment or environment knowledge.

### 2.3. Data-Driven Methods

The data-driven anomaly detection method can be used without having a priori knowledge such as the physical model of the system. That is, the monitored system data is analyzed to extract information about features and can be combined with historical data to diagnose anomalies in the system. This approach does not require extensive domain knowledge, relevant expert reasoning mechanisms, or establishment of accurate complex system models. This has become an important tool in the field of anomaly detection in the context of IoT and Industry 4.0. Traditional data-driven anomaly detection methods refer to anomaly detection for outliers and can be classified into four types, namely, statistical-based methods [11, 12], distance-based methods [13, 14], density-based methods [15, 16], and clustering-based methods [17, 18]. Statistical-based methods are model-based methods where a model is first created for the data and evaluated based on how well the object fits the model. For example, Laurikkala et al. [10] used box-line plots to identify outliers in the dataset while Kasliwal et al. [16] used a hybrid model of G-LDA which combines Latent Dirichlet Allocation (LDA) and genetic evolution techniques to detect anomalies in the network traffic. Distance-based methods consider a point anomalous if it is far from most of the points, because for the distance measurement, there are various ways. For example, Zhang et al. [17] used Mahalanobis distance for anomaly detection of hyperspectral images while Laxhammarand and Göran [19] used Hausdorff distance for phase dissimilarity measure for multidimensional trajectories of arbitrary length. Density-based methods consider outliers as objects that are in low-density regions. Density is commonly defined by proximity. Huang et al. [20] solved the problem of adaptive anomaly detection based on the Local Outlier Factor (LOF) algorithm while Celik et al. [21] discovered the anomalies present in temperature data based on the Density-Based Spatial Clustering of Applications with Noise (DBSCAN) algorithm. Clustering-based methods treat those data that do not belong to any class as anomalies by clustering them into classes. Münz et al. [22] proposed an anomaly detection approach based on the K-means algorithm for detecting anomalies in network monitoring data while Chitrakar and Chuanhe [23] proposed a hybrid method to solve the anomaly detection problem by combining Naïve Bayes classification and k-Medoids clustering method.

Since this paper aims to perform anomaly detection on a multi-sensor system based on extracting and analyzing potential correlations between features, the above methods do not match the goal of this paper.

### 2.4. Machine Learning Methods

As a typical representative of data-driven methods, machine learning can comprehensively analyze and mine potential anomalies in the high-dimensional time-series data generated by multi-sensor systems. Rauber et al. [24] designed a raw feature vector based on a set of statistical and signal characteristics and then used Support Vector Machine (SVM) to identify bearing faults. Chine et al. [25] calculated several feature parameters and used Artificial Neural Networks (ANN) for fault diagnosis of photovoltaic (PV) systems.

Although machine learning models, such as SVM [26], ANN [27], clustering algorithms [28], genetic algorithms [29], and fuzzy inference [30], can partially meet the needs of anomaly diagnosis and identification, changes in mechanical equipment load during operation can also affect the generalization ability of the models.

### 2.5. Deep Learning Methods

Deep learning is a branch of machine learning, and it can automatically discover features to meet the requirements of adaptive feature extraction for mechanical anomaly diagnosis. It effectively overcomes the shortcomings of traditional manual extraction of features, such as poor generalization ability and poor robustness, and reduces the uncertainty of traditional anomaly detection methods in the process of manual design and extraction. In recent years, different deep learning models, such as Deep Belief Networks (DBN), Stacked Autoencoder (SAE), Recursive Neural Network (RNN), and Conventional Neural Network (CNN), have received increasingly wide attention in intelligent anomaly detection [31]. Zhao et al. [32] proposed an approach for multi-sensor fault detection based on DBN, using deep learning models for the classification and prediction of sensor faults. Li et al. [33] used DBN for fault classification of bearings to reduce the manual operations in the detection process and to achieve intelligence in fault detection. Lei et al. [34] proposed a deep learning-based health monitoring method for mechanical equipment using frequency-domain signals as training data for Deep Neural Networks (DNN), completing adaptive feature extraction and intelligent health condition identification without the need for fault feature extraction through signal processing. Wan et al. [35] proposed a function-aware anomaly detection approach, in which both single function and short sequence patterns are considered as the function control characteristics, and a Wavelet Neural Network- (WNN-) based behavior model is established to detect function control misbehaviors caused by cyber intrusions in industrial automation. Kumar and Hati [36] proposed a CNN-based fault detection method for squirrel cage induction motor, in which small convolutional kernel and adaptive gradient optimizer were used to verify the performance of CNN model. Wilson et al. [37] proposed a deep learning-based fault diagnosis method for ship turbines using a deep bidirectional Long Short-Term Memory (LSTM) model for fast detection of turbines. Khorram et al. [38] investigated a Convolutional Recurrent Neural Network- (CRNN-) based fault diagnosis method, where the authors fused CNN and LSTM to form a Deep Neural Network for end-to-end fault diagnosis. Deng and Hooi [39] proposed an anomaly detection method based on GDN (Graph Deviation Network) in multivariate time series, in which the relationship between sensors is firstly found by GNN (graph neural network). The expected behavior of time series is then predicated, and the anomaly is finally identified by judging whether the predicted data violates the correlation. It considers the correlation as the scoring and judgment basis of whether the prediction data is abnormal.

In a nutshell, all the methods do not consider the potential correlation between features in a multi-sensor system, except for the method proposed in [39], which only uses the correlation as a threshold to judge whether the prediction data is abnormal and does not fully consider the dynamic and continuous characteristics of correlation to directly construct an anomaly detection model. In a multi-sensor system, sensors cooperate with each other to reflect the state of an entity, and the features used to describe the same entity are usually relevant. In general, the correlation is related to the physical characteristics of the entity, so the correlation is relatively stable, and if the correlation is broken, there may be an anomaly. Therefore, it is necessary to introduce correlation into anomaly detection for the purpose of finding potential anomalies caused by feature dependencies. Moreover, due to the time stability of the correlation, using the correlation between features which are obtained by time window for anomaly detection can effectively avoid the wrong detection results caused by discrete noisy data.

## 3. The Proposed Method

### 3.1. Correlation Characteristic of Multi-sensor System

Sensor data is generally considered to be the data collected by sensors for continuous sensing of the physical world. A multi-sensor system consists of several or even plenty of sensors. These sensors are used jointly to reflect the physical world. Except for continuity [40] and high dimensionality [41], the sensor data acquired by a multi-sensor system has several correlation characteristics, discussed as follows.

#### 3.1.1. Spatiotemporal Correlation

Sensor data is always applied to collect information about the physical world. Therefore, the sensor data can be correlated with the physical world it senses, especially in terms of time and space. In other words, there is a similarity between sensor data collected by similar sensors located in analogous time and space ranges. For example, multiple air quality monitors set up in the same environmental monitoring station at the same moment detect the same environmental indicators. Therefore, if sensor data is anomalous, the sensor data with which it has spatial-temporal correlation is more likely to be anomalous as compared to other sensor data [40].

#### 3.1.2. Data Similarity

If the sensor's monitoring objects are similar in behavior, the sensor data which is used to portray their behaviors should also have similarities. Take the power system as an example; the electricity meter data of household users with similar electricity consumption patterns are also approximately similar. It can be assumed that there are similarities between the data collected by similar sensors under similar behavioral, temporal, or spatial conditions. If sensor data is anomalous, the sensor data with which it has data similarity is more likely to be anomalous. That is, sensor data that disrupt data similarity may be anomalous. In a real-life production environment, monitoring data are relatively stable in most cases [42]. When a special event happens, the surrounding sensors usually monitor the situation and obtain data at the same time [43].

#### 3.1.3. Data Correlation

There may be correlations between the collected data of different sensors monitoring the same physical entity, including positive correlation and negative correlation. For example, the current and voltage collected by smart meters are correlated with power while the line loss is positively correlated with the current in the transmission system. When this correlation fluctuates, it may mark an anomaly of the physical entity; therefore, the correlation between different sensor data can also be used as a basis for determining anomalies. In fact, sensors always work together. Even if single sensor data is normal, it might be anomalous when it is calculated jointly with other sensor data [44].

### 3.2. Problem Statement

In a multi-sensor system, the training data usually consists of multiple sensors' data, that is, multivariate time-series data with *M* features from *N* sensors. The multivariate time-series data is expressed as the following formula:

(1) $$\begin{array}{c} S_{t,w}={\left(s^{1},s^{2},\ldots ,s^{m}\right)}^{T}ɛR^{m\times w}. \end{array}$$

In (1), *t* denotes the current timestamp, *w* denotes the size of the time window, and *m* denotes the feature dimension of the temporal data. Moreover, *s*^*i*^ (*i* *=* 1,2,…, m) denotes the column vector composed of the values of feature *i* in the dataset over the time window from timestamp *t* to timestamp *t* *+* *w-*1, and *S*_*t,w*_ denotes a fragment of the time series. Using *s*^*k*^*=*(*s*^*k*^_*t*_*, s*^*k*^_*t+1*_*,…, s*^*k*^_*t*_ _*+*_ _*w-*1_)^*T*^ ∈ *R*^w^ denotes the value vector of the *k*th feature within a time window.

The goal of this paper is to identify anomalies in physical entities characterized by multivariate time-series data generated by the same sensors but over a different time slice. The output of the proposed method is a group of labels used to show the result of anomaly detection for each time window; i.e., label_*t*_ ∈ {−1, 1}, in which label_*t*_ = 1 indicates that the time window *t* is normal, and label_*t*_ = −1 is the opposite.

### 3.3. Method Overview

**Figure 1:**
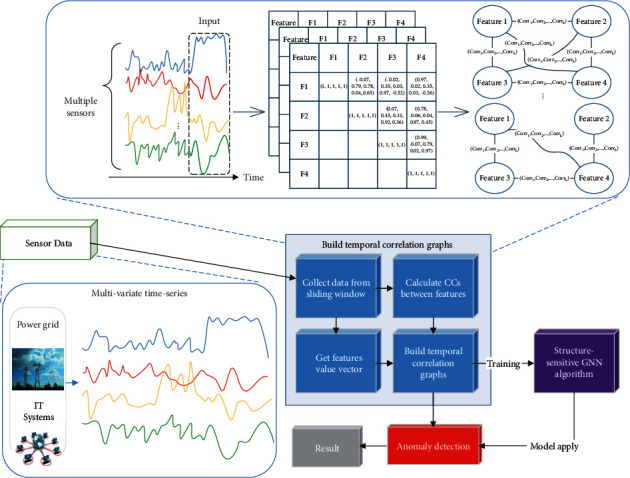
Overview of the proposed method.

The goal of the proposed correlation-based anomaly detection method is to capture the temporal correlations between sensors and then identify whether the normal temporal correlation patterns between sensors are violated. As shown in Figure 1, the proposed method mainly involves two parts, namely correlation-based graph model construction and graph-based anomaly detection.

#### 3.3.1. Correlation-Based Graph Model Construction

It represents the unique characteristics of the multiple sensors for each time window with multivariate time-series data, and these multidimension time series are converted into a set of graph structures.

#### 3.3.2. Graph-Based Anomaly Detection

It trains an anomaly detection model with a structured-sensitive GNN and then identifies deviations from the learned temporal correlations.

### 3.4. Correlation-Based Graph Model Construction

Data captured by a multi-sensor system are represented as multivariate temporal data; i.e., there are temporal correlations between different sensors. To represent and analyze the correlations, these multidimensional time-series data can be transformed into a set of temporal correlation graphs *G* = (*V*, *E*) according to the time window. A temporal correlation graph is an undirected graph, where the nodes represent features in the multi-sensor systems while the edges represent the correlation between these features.

#### 3.4.1. Construction of Nodes

Each feature of the multi-sensor system is considered as a node in the temporal correlation graph, and the node information consists of *s*^*i*^*=*(*s*^*i*^_*t*_*, s*^*i*^_*t+1*_,…*, s*^*i*^_*t*_ _*+*_ _*w-*1_)^*T*^, where *i* *=* 1, 2,…,*m*, where *m* is the total number of nodes.

#### 3.4.2. Construction of Edges

The edges in a temporal correlation graph are defined as the temporal correlations between nodes. Take the example of constructing an edge between node *V*_*i*_ and node *V*_*j*_; the edge is depicted as a row vector *e* = (*corr*_*1*_*, corr*_*2*_,…*, corr*_*k*_) consisting of *k* kinds of correlation coefficients between node *V*_*i*_ and *V*_*j*_ on the time series *S*_*t,w*_. To comprehensively measure the correlation between nodes, this study fuses multiple correlation coefficients to constitute the edge information, including Manhattan distance, Euclidean distance, Chebyshev distance, Pearson correlation coefficient, and Spearman correlation coefficient.

**Figure 2:**
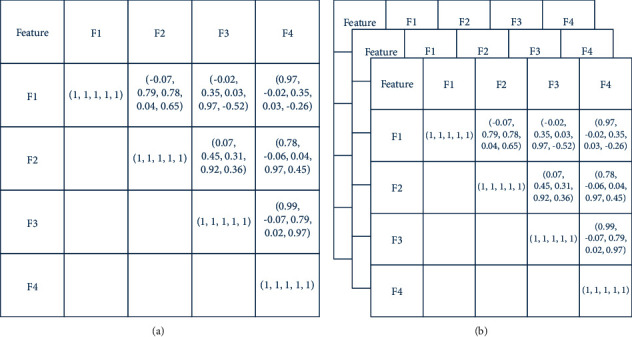
Temporal correlation matrices: (a) inter-feature correlation coefficient matrix; (b) multidimensional temporal correlation matrices.

By calculating the inter-feature correlation coefficients of each pair of features, the corresponding inter-feature correlation coefficient matrix for a time window can be obtained, as shown in Figure 2(a). If the inter-feature correlation coefficient matrices of continuous-time windows are combined, a group of multidimensional temporal correlation matrices are built, as shown in Figure 2(b).

#### 3.4.3. Construction of Temporal Correlation Graphs

**Figure 3:**
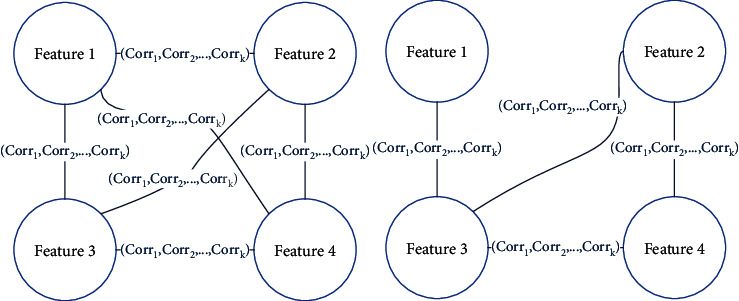
Examples of temporal correlation graphs.

To identify the fluctuation of correlations between features, the proposed method converts each matrix array into a temporal correlation graph. Subsequently, the correlation strength between pairs of features is calculated according to the correlation coefficient. Then, the value of correlation coefficient is further analyzed; if it is within the threshold of the correlation coefficient in the normal mode, the edge is reserved; otherwise, the edge is removed from the graph. That is, a temporal correlation graph can be an incomplete graph, and the structures of different temporal correlation graphs may not be the same, as shown in Figure 3.

When the multidimensional time series are converted into a set of temporal correlation graphs, these graphs are used as an input for the graph-based deep learning algorithm. Besides, the proposed method is still a supervised one; the correlations among features always have high stability. Since anomalies in a multi-sensor system are usually reflected as non-single outliers and the significant fluctuations in the correlations between features in different time windows may exist, a label is assigned to each temporal correlation graph, which is used to mark whether the time series is abnormal or not. If anomalies exist, the graph label is set to positive; otherwise, it is set to negative.

### 3.5. Graph-Based Anomaly Detection

The method uses temporal correlation graphs to represent features and the correlations between them. Changes in the graph structure, point, and edge attributes can reflect changes in the degree of dependency between features. Therefore, an anomaly detection of multi-sensor systems can be converted into a binary classification problem based on the GNN model.

#### 3.5.1. Construction of Graph-Based Anomaly Detection Model

**Figure 4:**
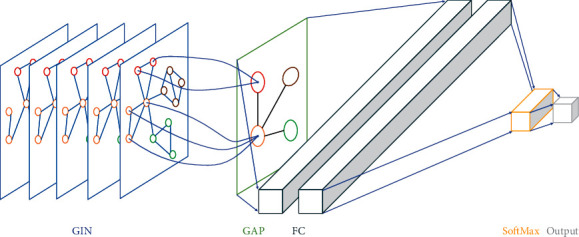
Network architecture.

Figure 4 shows the network architecture of the anomaly detection model.

The correlation between features is also a kind of time-series data, whose fluctuation is universal. In this paper, the edges of temporal correlation graph are conducted based on the strength of correlation between features. If the correlation is very low or even does not exist, there is no edge between features. That is, the temporal correlation graphs are heterogeneous. Therefore, a structure-sensitive graph convolution neural network, GIN (Graph Isomorphism Network), is applied to the temporal correlation graphs by encoding feature and the correlation between features according to the requirements of GIN. Then, Global Add pooling (GAP) which refers to the default pooling function global_add_pool in torch_geometric maps the features of different samples to the same size and the pooled features are output to the Softmax classifier for classification after being mapped to the sample label space by the fully connected (FC) layer. In addition, for this model, cross-entropy loss is selected as the training loss function, and Adam is used to optimize the neural network.

#### 3.5.2. Anomaly Detection

**Figure 5:**
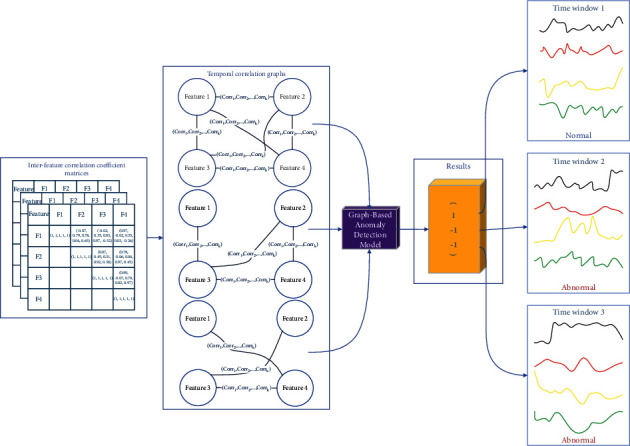
The workflow of the graph-based anomaly detection.

Figure 5 shows the workflow of graph-based anomaly detection. The multidimensional time series is firstly transformed into a set of inter-feature correlation matrices, and then the corresponding temporal correlation graphs are established. The graph-based anomaly detection model treats these graphs as input and produces output based on the binary classification.

## 4. Evaluation

### 4.1. Experimental Setting

Dataset: In order to evaluate the validity of the proposed method, this paper conducted extensive experiments on three industrial datasets. These datasets are described in detail as follows:

Dataset 1: Air Quality1 is from the air quality online detection and analysis platform (https://www.aqistudy.cn/historydata/). The platform contains air quality and meteorological data collected hourly in more than 300 cities from December 2013 to December 2021. Dataset 1 selected the historical data from January 2014 to December 2018 in Air Quality1, including AQI, PM_2.5_, PM_10_, CO, NO_2_, SO_2_, and O_3_. Among them, AQI is used to measure air quality based on the standard of “Technical Regulation on Ambient Air Quality Index (on trial)” [45]. The data description of other features in Dataset 1 is shown in Table 1, in which the unit of each feature is *μ*g/m^3^.

Dataset 2: Air Quality2 is from Kaggle (https://www.kaggle.com/amritpal333/tps-july-2021-original-dataset-clean). The installation is in a heavily polluted area of an Italian city, and the data are collected through an embedded air quality chemical multi-sensor unit containing five metal oxide sensors' data and other relevant data. The five metal oxide sensors' data refer to carbon monoxide, total nitrogen oxides, non-methane hydrocarbons, NO_2_, and benzene. Air Quality2 is accumulated according to the “Ambient Air Quality Standards” [46]. The dataset contains six kinds of air quality data from March 2004 to February 2005, with data collected hourly. After data cleaning, a total of 827 time points were included. To ensure the scale, Dataset 2 was constructed by replicating Air Quality2, and the expanded dataset contains data of 414327 time points. The data description of Dataset 2 is shown in Table 2, in which the units are, respectively, mg/m^3^, *μ*g/m^3^, *μ*g/m^3^, °C, %, and g/m^3^.

Dataset 3: The gas chromatography data was obtained from the industrial dataset of power enterprises, consisting of the dissolved gas content of insulating oil of oil-filled power equipment. It is the basis for power generation and supply enterprises to judge whether there are latent overheating, discharge, and other faults of oil-filled power equipment in operation. Moreover, it is also the necessary data for oil-filled electrical equipment manufacturers to carry out factory inspection of their equipment. Dataset 3 is time-series data consisting of transformer oil chromatography data, containing six data items, namely, H_2_, CH_4_, C_2_H_4_, CO, C_2_H_6_, and total hydrocarbons, containing 20128 time points. Based on the “Guide to the Analysis and the Diagnosis of Gases Dissolved in Transformer Oil” [47] issued by the National Energy Administration, if the gas content data exceeds the threshold value, there may be an anomaly, and if multiple gas contents are consistently abnormal, the transformer can be judged to be faulty. The data description of Dataset 3 is shown in Table 3, in which the unit of each feature is *μ*L/L.

In the field of anomaly detection, if the percentage of negative samples is low, it will affect the quality of the fault model and influence the detection effect. In addition, to fully evaluate the validity of the proposed method, it is essential to conduct experiments on datasets with different anomaly distributions. Therefore, a data enhancement algorithm whose framework is described in Algorithm 1 is used to inject anomalies into the original data. The method of data augmentation is as follows: For each passing step, an attribute is selected, and the original time-series data is modified by the data augmentation sequence array within the data augmentation window.

The distribution of anomalies for the datasets is listed in Table 4.

Baselines: The following algorithms are selected for comparison for the sake of verifying the effectiveness of the proposed method. The detailed description of these comparison algorithms is as follows.

Decision Tree: The purpose of decision trees is to build a model which can forecast the value of a specified variable according to a set of decision rules extrapolated from the data characteristics. It can be used as a nonparametric supervised algorithm for classification.

Logistic Regression: Logistic regression is used to evaluate the possibility of a particular event by settling binary classification problems through the machine learning method.

Naïve Bayes: As a supervised algorithm, Naïve Bayes is a simple and effective classification algorithm based on Bayes' theorem.

Quadratic Discriminant Analysis: Quadratic Discriminant Analysis (QDA) is a classifier that makes classifications according to the difference in covariance, which is particularly useful for many features.

Support Vector Machine: Support Vector Machine (SVM) is a binary classification algorithm that maps the feature vector of each entity to a point in the space and classifies these apace points by finding an optimal separating hyperplane.

Convolutional Neural Networks: As a deep learning model, Conventional Neural Network (CNN) is a feed forward neural network based on convolution calculation, which can classify the input information according to the hierarchical structure.

Evaluation Metrics: Although anomalies are common, the proportion of anomalies in a dataset is not high, so most of the data used for anomaly detection are unbalanced data. In the classification problem of balanced data, accuracy is a common evaluation metric. In the classification of unbalanced data, F1 score and AUC (Area Under Curve) score are common evaluation metrics. Thus, F1 score and AUC score are used to measure the performance of the proposed method.

F1 Score: Precision focuses on evaluating the proportion of real positive data in all data predicted as positive, while recall focuses on assessing how much of all the positive data has been successfully predicted as positive. F1 score is a harmonic mean of precision and recall. In general, the value of F1 score will be high only when both precision and recall are good.

AUC Score: Facing the unbalanced data, ROC (Receiver Operating Characteristic) curve ignores the imbalance of samples and only considers the classification ability of the model. That is, when the proportion of positive and negative samples changes, the discrimination ability of the model remains unchanged, and the shape of ROC curve will not change. However, ROC is not intuitive enough, so AUC which refers to the area under the ROC curve becomes a way to reflect the classification ability expressed by the ROC curve. Since AUC score is robust to the data with heterogeneous distribution of positive and negative samples, this paper uses AUC score as a metric for performance evaluation, and the larger the AUC, the better the effect.

Environments: A XenServer virtual machine is used to conduct these experiments, and its configuration is as follows: CentOS7.3, one Intel Core i5-1135G7 processor, 16.00 GB RAM, 500 GB hard disk. The proposed method and the baselines are all implemented in Python and PyTorch 1.6.0.

Hyperparameter Settings: The model in this paper involves five hyperparameters, which are *window_size*, *learning_rate*, *batch_size*, correlation coefficient-related parameter *threshold_1*, and feature value anomaly-related parameter *threshold_2*. *window_size* is the size of the sliding window, which can collect data on the time series according to the specified length; *learning_rate* and *batch_size* are the parameters used to train the deep learning model; *threshold_1* is the upper limit of the inter-feature correlation coefficient fluctuation; *threshold_2* is the upper limit of feature value anomalies within the sliding window.  Table 5 gives the setting of these hyperparameters in the experiments.

In addition, the model setting for the structure-sensitive GNN is as follows: the model was trained for 300 epochs with a learning rate of 0.01 and a batch size of 10.

### 4.2. Experiments

#### 4.2.1. Experiment on Accuracy

The mean and standard deviation are two relevant statistical indicators whose combined use can describe the overall characteristics of the data more comprehensively. The former indicator shows the concentration tendency of the data, while the latter indicator shows the off-center tendency of the data. In other words, the smaller the standard deviation, the better the representativeness of the mean.  Table 6 shows the accuracy of anomaly detection by listing the mean and standard deviation of F1 scores of the proposed method and the baselines in three datasets.

As shown in Table 6, the standard deviation values of the F1 score in the three datasets are, respectively, 0.006, 0.031, and 0.004. The two standard deviation values of CNN are 0.001 lower than or equal to those of the proposed method, while the other 16 values are not as good as those of the proposed method. This result shows that the F1 score of the proposed method is more concentrated and the proposed method is more stable. The mean values of the F1 score in the three datasets are, respectively, 0.92, 0.90, and 0.95. Among all the mean values of F1 score, only the value of the decision tree on Dataset 3 is 0.02 higher than that of the proposed method. This proves that the proposed method outperforms all the other baselines. In addition, all the three mean values of the F1 score of the proposed method are more than 0.90, while the baselines failed to do so. Overall, the F1 score of the proposed method is low for off-center tendency and high for concentration tendency. F1 score is a comprehensive evaluation of the precision and recall of a method. The results indicate that the method proposed in this paper achieves well balanced precision and recall, which shows that both precision and recall are good.

#### 4.2.2. Experiment on Classification Ability

In Table 7, the classification ability of different anomaly detection methods in terms of the mean value and standard deviation of AUC score on the three datasets is given.

As shown in Table 7, the standard deviation values of the AUC score in the three datasets are, respectively, 0.005, 0.009, and 0.005. Only three standard deviation values are, respectively, 0.001, 0.001, and 0.002 lower than those of the proposed method, while the other 15 values are not as good as those of the proposed method. This result indicates that the mean value of the AUC score of the proposed method is quite concentrated. The mean values of the AUC score in the three datasets are, respectively, 0.96, 0.98, and 0.95. Except that the value of the decision tree on Dataset 3 is 0.01 higher than that of the proposed method, the proposed method outperforms all the baselines. The mean values of the AUC score of the proposed method on the three datasets all exceed 0.95, while the other methods do not reach this value. AUC score reflects the classification ability of the models. Therefore, the results illustrate that the method proposed in this paper is superior to the baselines in the classification ability on all the three datasets no matter whether the datasets are balanced or not.

In view of the above experiments, it is found that the method based on the neural network has better performance. Meanwhile, the decision tree method also has good results on Dataset 3. The reason is that Dataset 3 is the actual measurement data of the substation equipment of the National Grid, and the original dataset does not contain anomalies, while the injected anomalies are more obviously different from the normal data. Since the decision tree uses a tree structure, it is easier to decide if the two categories are completely different when binary classification is performed, so its classification results become better. However, the F1 score and AUC score of the decision tree in Dataset 1 and Dataset 2 are not as good as the proposed method. That is, the stability of the decision tree is lower than that of the proposed method. In brief, the experimental results of the proposed method on all three datasets illustrated that the method introduced in this paper is a better classifier than the baseline methods by combining the two metrics, especially for the anomaly detection problem in which unbalanced data may exist.

## 5. Conclusion

For the anomaly detection problem of multi-sensor systems, this paper proposes an anomaly detection method which innovatively makes use of the correlation between features and transforms the anomaly detection of multivariate time-series data into graph classification problem. Since the correlation of features fluctuates with time, the concept of temporal correlation graph is firstly proposed, and the method in which both the feature and the correlation between features are, respectively, encoded into nodes and edges is given to construct the temporal correlation graph. Subsequently, the graph classification model is established for the constructed graph structure data which may have structural differences by the structured-sensitive GNN. Finally, the anomalies of the multi-sensor system are identified by determining whether the graph data are anomalous. The results of the experiments show that the mean values of F1 score and AUC score of the proposed method exceed 0.90 and 0.95, respectively, which are better than those of other baseline methods. That is, the proposed method achieves well balanced precision and recall and is a better classifier which provides better discrimination. Therefore, the proposed method can effectively identify the physical entity anomalies reflected by multidimensional time series in multi-sensor systems. Future research will focus on conducting more abundant experiments to analyze the influence of graph-related attributes and labels on the classification effect and to study more domain-targeted anomaly detection methods for multi-sensor systems.

## Figures and Tables

**Algorithm 1:**
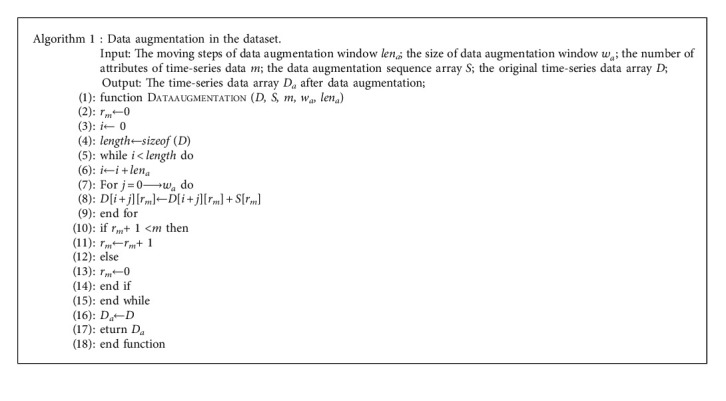
Framework of data augmentation in the dataset.

**Table 1:** Data description of Dataset 1.

Feature	Range	Threshold
PM_2.5_	0∼885.0	75
PM_10_	0∼1700.0	150
SO_2_	0∼429.0	150
CO	0∼10.4	4
NO_2_	0∼183.0	80
O_3_	0∼311.0	160

**Table 2:** Data description of Dataset 2.

Feature	Range	Threshold
CO	0.3∼8.1	4
NO_x_	12.0∼478.0	100
NO_2_	19.0∼196.0	80
Temperature	6.30∼30.00	-
Relative humidity (%)	14.90∼83.20	-
Absolute humidity	0.4023∼1.4852	-

**Table 3:** Data description of Dataset 3.

Feature	Range	Threshold
H_2_	1.357∼171.160	150
CH_4_	0.042∼30.183	-
C_2_H_4_	0∼15.082	-
C_2_H_2_	0∼15.000	5
CO	12.996∼1202.545	-
Total hydrocarbon	2.242∼161.771	150

**Table 4:** Detailed information of datasets in the experiments.

Dataset	Positive samples	Negative samples	Negative ratio (%)
Dataset 1	4142	6807	37.82
Dataset 2	1562	256	85.90
Dataset 3	898	3126	22.30

**Table 5:** Settings of hyperparameters.

Hyperparameter	Dataset 1	Dataset 2	Dataset 3
window_size	5	5	5
threshold_1	0.25	0.25	0.05
threshold_2	2	2	3
learning_rate	0.01	0.01	0.01
batch_size	10	10	10

**Table 6:** Anomaly detection accuracy in terms of F1 score, on three datasets.

Method	Dataset 1		Dataset 2		Dataset 3	
	Mean	Standard deviation	Mean	Standard deviation	Mean	Standard deviation
Logistic regression	0.87	0.007	0.82	0.035	0.94	0.006
Decision tree	0.91	0.006	0.83	0.086	0.97	0.011
Naïve Bayes	0.89	0.008	0.70	0.042	0.86	0.017
QDA	0.88	0.027	0.86	0.066	0.87	0.021
SVM	0.52	0.008	0.40	0.047	0.82	0.032
CNN	0.90	0.005	0.86	0.027	0.95	0.005
Proposed method	**0.92**	**0.006**	**0.90**	**0.031**	**0.95**	**0.004**

Bold font is to highlight the results of the proposed method.

**Table 7:** Classification ability of anomaly detection in terms of AUC score, on three datasets.

Method	Dataset 1		Dataset 2		Dataset 3	
	Mean	Standard deviation	Mean	Standard deviation	Mean	Standard deviation
Logistic regression	0.90	0.006	0.98	0.008	0.95	0.008
Decision tree	0.90	0.006	0.89	0.051	0.96	0.012
Naïve Bayes	0.93	0.007	0.96	0.010	0.84	0.025
QDA	0.92	0.009	0.91	0.030	0.92	0.020
SVM	0.75	0.012	0.81	0.022	0.68	0.042
CNN	0.96	0.004	0.96	0.007	0.93	0.006
Proposed method	**0.96**	**0.005**	**0.98**	**0.009**	**0.95**	**0.005**

Bold font is to highlight the results of the proposed method.

## Data Availability

Dataset 1 and Dataset 2 used to support the findings of this study are included within the article. Dataset 3 used to support the findings of this study was supplied by China State Grid under license and so cannot be made freely available. Requests for access to these data should be made to the corresponding author for an application of joint research.
